# Covid 19-consideration on separation and mourning ritual

**DOI:** 10.1192/j.eurpsy.2021.853

**Published:** 2021-08-13

**Authors:** S. Cerino, A. Amati

**Affiliations:** 1 Sphere, ECOS - EU, Massa Martana, Italy; 2 Psychiatry Department, University of Magna Graecia, Naples, Italy

**Keywords:** Mourning, Ritual craing, Grief, covid 19 emergency

## Abstract

**Introduction:**

During COVID 19 epidemic it has been interesting to observe the gradual transformation of the daily routine into death, sorrow and pain. The moment of transition from life to death was really changed by distressing for survivors who had to face with unexpected ways to live their mourning.

**Objectives:**

The paper would like to analyze the mounring ritual during COVID 19 epidemic.

**Methods:**

During COVID 19 tragedy victims and survivors have been first associated by the infection and then dramatically separated by its effects. It has been necessary to re-relaborate new procedures of separation from deceased, as far for laws prohibitions it was no longer possible to use the traditional ones.

**Results:**

So a leaving “ritual” re-emerged very similar to the “crying ritual” of the Southern Italy folkloric culture. The traditional wailers have been substitued by the windows flash mobs trying to replace the forced absence of “pietas”, with a moment of positive sharing of physical distance between life and death, using sounds, songs, tools that always, in farming culture help to exorcise and take away death.

**Conclusions:**

The relevant starting powerlessness to face the fast disease diffusion, its intrinsic seriousness, inspired surprising capabilities of immediate reaction and active mobilization in response to the attack suffered by Koerper and Leib (in Heidegger sense) which actualized in the research of a new “separation” dimension. In the end, as psychiatrists, we have to notice how this collectivization practice is actually a big distress container and wonder where and how this distress will finally arrive

